# Eosinophilic pediatric ABPM lacking fungal sensitization responsive to anti–IL-5

**DOI:** 10.1016/j.jacig.2025.100612

**Published:** 2025-11-20

**Authors:** Mari Mizutani, Takayasu Nomura, Akiko Nakaoka, Hisashi Tanida, Yoshihiro Kanemitsu, Keinosuke Hizuka, Shigeharu Ueki, Shinji Saitoh

**Affiliations:** a Department of Pediatrics and Neonatology, Nagoya City University Graduate School of Medical Sciences, Nagoya, Japan; b Department of Respiratory Medicine, Allergy and Clinical Immunology, Nagoya City University Graduate School of Medical Sciences, Nagoya, Japan; c Department of General Internal Medicine and Clinical Laboratory Medicine, Akita University Graduate School of Medicine, Akita, Japan

**Keywords:** Allergic bronchopulmonary mycosis, eosinophilic airway inflammation, high-attenuation mucus, Charcot-Leyden crystals, anti–IL-5 therapy, pediatric asthma

## Abstract

A pediatric patient with allergic bronchopulmonary mycosis with high-attenuation mucus and no fungal sensitization achieved remission with anti–IL-5 therapy, highlighting the diagnostic value of high-attenuation mucus and the role of eosinophilic inflammation in non–IgE-dominant airway disease.

Allergic bronchopulmonary mycosis (ABPM) and allergic bronchopulmonary aspergillosis (ABPA) are eosinophilic airway diseases caused by hypersensitivity to fungi colonizing the bronchi. According to recent evidence and updated definitions, ABPA refers to *Aspergillus*-associated disease, whereas ABPM includes other fungi.^1^ Their pathophysiology comprises 3 key elements: (1) airway colonization by fungi without invasion, (2) types I and III hypersensitivity to fungal antigens, and (3) eosinophilic airway inflammation. These components underlie the disease spectrum and are central to diagnostic and therapeutic approaches.

According to the revised International Society for Human and Animal Mycology (ISHAM) criteria, high-attenuation mucus (HAM) observed on chest computed tomography is regarded as a pathognomonic radiologic finding and may alone confirm ABPA and/or ABPM even if other diagnostic components are missing.^2^ This important revision has broadened the disease spectrum to include patients lacking serologic evidence of fungal sensitization.

Here, we report a pediatric case of ABPM that fulfilled the revised ISHAM criteria based on asthma, peripheral eosinophilia, and HAM despite the absence of detectable fungal colonization or sensitization. Histologic analysis of expectorated mucus demonstrated Charcot-Leyden crystals (CLCs) and eosinophil cytolysis, supporting an eosinophil-driven pathophysiology.^3^^,^^4^ To our knowledge, this case represents the youngest Japanese patient with HAM-positive ABPM,^5^ and it underscores a non–IgE-dominant, eosinophil-driven disease phenotype that responded to anti–IL-5 therapy.

## Case report

**Fig 1:**
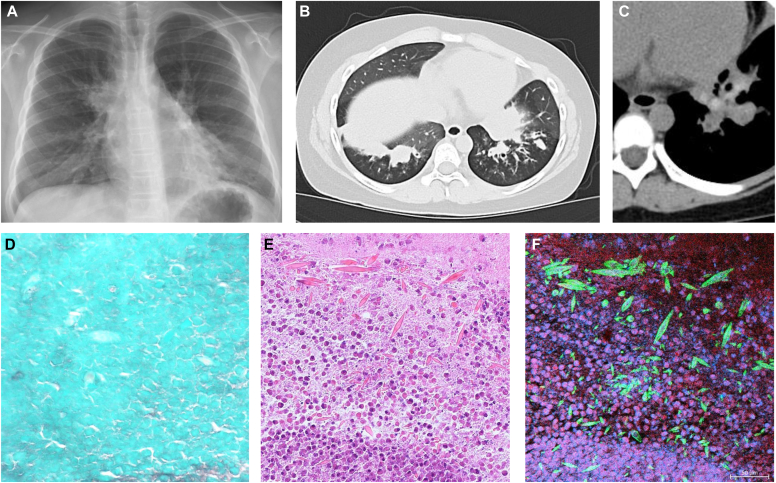
Radiologic and histologic findings. **A-C,** Chest radiography and computed tomography reveal central bronchiectasis, mucoid impaction, and HAM. **D,** Grocott staining shows no fungi. **E,** Hematoxylin and eosin staining reveals eosinophils and Charcot-Leyden crystals. **F,** Immunostaining confirms galectin-10–positive (*green*) crystals and MBP-positive (*red*) cytolytic eosinophils lacking cytoplasmic galectin-10. DNA was stained with Hoechst 33342 (*blue*).

A 9-year-old Japanese girl with moderate persistent asthma presented with progressive wheezing and dyspnea. She was afebrile (body temperature 37.3°C) and hypoxemic (peripheral capillary oxygen saturation [Spo_2_] level 91%). Chest radiography showed perihilar bronchial wall thickening (Fig 1, *A*). High-resolution computed tomography demonstrated central bronchiectasis and mucoid impaction (Fig 1, *B*), with HAM in the lower lobes exceeding the density of the adjacent muscle (Fig 1, *C*).^2^

Initial blood tests revealed eosinophilia (1404 eosinophils/μL) and mildly elevated level of total IgE (233 IU/mL). Fungus-specific IgE testing was performed at a commercial reference laboratory (SRL, Tokyo, Japan) using a fluorescence enzyme immunoassay (ImmunoCAP, Thermo Fisher Scientific, Waltham, Mass). Levels of both *Aspergillus fumigatus* and a fungal mix (*Penicillium*, *Cladosporium*, *Candida*, *Alternaria*, and *Helminthosporium*) were below the detection limit. Serum *Aspergillus fumigatus*–specific IgG was likewise undetectable when measured by ELISA.

A thick mucus plug was expectorated a few days after the patient had started taking oral corticosteroids and analyzed. Cultures and Grocott staining were negative for microbial elements (Fig 1, *D*). Hematoxylin and eosin staining showed dense eosinophilic infiltration with numerous needle-like crystals (Fig 1, *E*). Double-immunostaining for eosinophil major basic protein (MBP) and galectin-10 confirmed the crystals as CLCs (Fig 1, *F*). Staining also revealed cytolytic eosinophils with cytoplasmic galectin-10 release and diffuse extracellular MBP deposition. On the basis of asthma, HAM, eosinophilia, and eosinophilic mucus pathology, a diagnosis of ABPM was made under the revised ISHAM criteria.^2^

**Fig 2:**
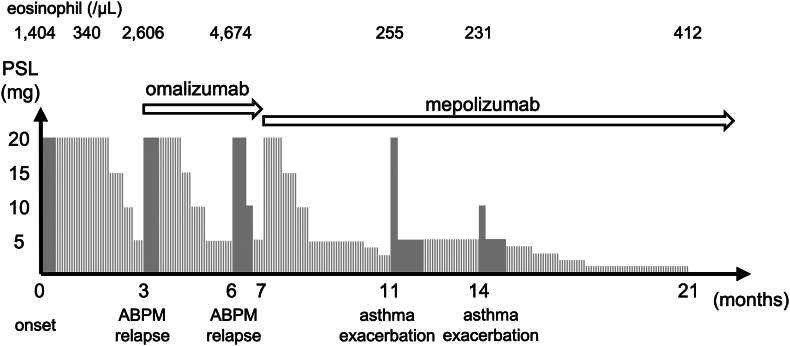
Graphical representation of the patient’s clinical course showing eosinophil counts, corticosteroid dosing, and biologic therapies. *Gray bars* indicate daily prednisolone (PSL); *striped bars* indicate alternate-day dosing. ABPM relapses were associated with eosinophilia. Events labeled asthma exacerbation reflect dyspnea without eosinophilia, which is attributed to coexisting asthma rather than to ABPM.

Treatment with oral prednisolone (20 mg per day) initially improved the patient’s symptoms and reduced her eosinophil count to 340 cells/μL. However, 2 relapses occurred during tapering. At 3 months, her eosinophil count rose to 2606 cells/μL with symptom recurrence. Omalizumab therapy was initiated at 4 months, but a relapse occurred at 6 months, with the patient’s eosinophil count reaching 4674 cells/μL (Fig 2). Mepolizumab (100 mg every 4 weeks) was started at month 7, leading to sustained remission, resolution of dyspnea, and stable eosinophil counts (<300 cells/μL). Steroids were successfully discontinued. Over the following 15 months, the patient remained relapse-free, with her asthma considered well controlled despite occasional mild symptoms and her eosinophil count remaining stable (231-412 cells/μL) (Fig 2).

## Discussion

This case provides several important insights into ABPM diagnosis and management in pediatric patients. First, HAM had decisive diagnostic value. The 2024 revised ISHAM guidelines designate HAM as pathognomonic for ABPA and/or ABPM, which is sufficient for diagnosis even in the absence of fungal sensitization and colonization.^2^ Our patient met this radiologic criterion, making ABPM highly likely despite negative serologic and culture results.

The histologic presence of CLCs and cytolytic eosinophils further supports the concept of eosinophilic mucus disease, which is a recently proposed entity encompassing ABPM and related disorders.^3^^,^^4^ These findings reflect eosinophil-mediated pathology and align with the notion of “luminal hypereosinophilic disease.”^6^ Galectin-10 crystallization with CLC formation is increasingly recognized not only as a marker of eosinophil activation but also as a contributor to mucus viscosity, impaired clearance, and persistent airway inflammation.^3^

The pediatric presentation of HAM-positive ABPM is exceedingly rare. A nationwide Japanese survey reported no cases in individuals younger than 15 years, with a median age of onset of 57 years.^5^ Thus, this patient is likely the youngest Japanese case patient with HAM-positive ABPM. The absence of fungal involvement raises a critical issue, namely, whether HAM formation can result solely from severe eosinophilic inflammation. Although the revised ISHAM criteria allow diagnosis in such scenarios, further studies are needed to clarify the specificity of HAM for diagnosis of ABPM.

Regarding therapy, systemic corticosteroids remain the first-line treatment according to the 2024 ISHAM statement, with antifungals considered adjunctive therapy (mainly in *Aspergillus*-associated disease). Evidence for antifungals in non-*Aspergillus* ABPM or pediatric patients is limited. Given our patient’s negative fungal testing result, young age, and steroid-dependent eosinophilic disease, we prioritized eosinophil-targeted biologics over antifungals. Mepolizumab achieved durable remission and steroid withdrawal, contrasting with relapse during omalizumab therapy. This course highlights the central role of eosinophilic inflammation, rather than solely IgE-mediated allergy, in certain ABPM phenotypes.^1^^,^^7^ Recent reports have also described benefit from benralizumab in mepolizumab-refractory cases,^8^ reinforcing the rationale for IL-5–targeted therapy. Real-world studies continue to support anti–IL-5 biologics as effective for ABPA and/or ABPM.^9^

Thus, phenotype-guided therapy is crucial: whereas corticosteroids control acute inflammation, biologics tailored to the dominant immunopathology may secure long-term remission. This case emphasizes the fact that anti–IL-5 therapy can be particularly beneficial in eosinophilic, non–IgE-dominant ABPM.

## Conclusion

This case illustrates a rare pediatric presentation of ABPM with HAM and eosinophil-dominant pathology in the absence of fungal sensitization. It demonstrates the diagnostic utility of HAM under the revised ISHAM criteria and highlights eosinophilic inflammation as a key mechanism in mucus plugging. The favorable response to anti–IL-5 therapy supports phenotype-based treatment approaches. Importantly, this case raises the question of whether HAM can arise without fungal involvement, which is a matter requiring further investigation regarding its specificity.

Declaration of generative artificial intelligence and artificial intelligence–assisted technologies in the writing process. During the preparation of this work, the authors used ChatGPT (based on GPT-5 [OpenAI, San Francisco, Calif]) to improve readability and language. After using this tool, the authors reviewed and edited the content as needed and take full responsibility for the content of the article.

## Disclosure statement

Supported in part by Japanese Society for the Promotion of Science Grants-in-Aid for Scientific Research Program (grants 24K11593 and 25K11699), the Japan Agency for Medical Research and Development (grant focusing on allergic disease and immunology JP25ek0410138), the Key Research Laboratory Fund, and the Environmental Restoration and Conservation Agency (to S.U.).

Disclosure of potential conflict of interest: The authors declare that they have no relevant conflicts of interest.
